# The role of dissociation in ketamine’s antidepressant effects

**DOI:** 10.1038/s41467-020-20190-4

**Published:** 2020-12-22

**Authors:** Elizabeth D. Ballard, Carlos A. Zarate

**Affiliations:** Section on the Neurobiology and Treatment of Mood Disorders, Intramural Research Program, National Institute of Mental Health, National Institutes of Health, Bethesda, MD 20892 USA

**Keywords:** Drug discovery, Neuroscience, Biomarkers

## Abstract

Ketamine produces immediate antidepressant effects and has inspired research into next-generation treatments. Ketamine also has short term dissociative effects, in which individuals report altered consciousness and perceptions of themselves and their environment. However, whether ketamine’s dissociative side effects are necessary for its antidepressant effects remains unclear. This perspective examines the relationship between dissociative effects and acute and longer-lasting antidepressant response to ketamine and other N-methyl-D-aspartate (NMDA) receptor antagonists. Presently, the literature does not support the conclusion that dissociation is necessary for antidepressant response to ketamine. However, further work is needed to explore the relationship between dissociation and antidepressant response at the molecular, biomarker, and psychological levels.

## Abstract (web summary)

Ketamine is associated with rapid antidepressant effects and temporary dissociative experiences, and this review examines whether these dissociative symptoms are necessary for antidepressant efficacy. Although the current literature does not support this relationship, further work is needed to explore possible associations at the molecular, biomarker, and psychological levels.

## Introduction

The onset of antidepressant treatment response has recently been proposed to occur in three time periods: an immediate-response window of 1 to 2 days; a rapid-response window of up to 1 week; and a slow-response window of more than 1 week^1^. In this context, the N-methyl-D-aspartate receptor (NMDAR) antagonist (*R,S*)-ketamine (ketamine) and other next-generation rapid-acting antidepressants (RAADs) represent a new frontier for psychiatric research. Traditional antidepressants have a lag of onset that lasts weeks to months (i.e., a slow-response window) and limited efficacy for treatment-resistant depression (TRD) and bipolar depression. In contrast, ketamine is associated with rapid improvements in depressive symptoms that occur within minutes to hours (i.e., an immediate-response window), with the largest effect sizes observed within 1 day^2^.

Ketamine was originally synthesized as an alternative to phencyclidine (PCP)^3^. However, ketamine has less potent psychotomimetic and dissociative effects than PCP, a wider anesthetic safety window, and a shorter half-life^3^. Over the last half-century, it has been in widespread, global use as an anesthetic. Although its immediate antidepressant effects were only observed two decades ago^4^, the literature has since expanded with evidence of its efficacy in individuals with TRD^2,5–7^, bipolar depression^8,9^, post-traumatic stress disorder (PTSD)^10^, obsessive-compulsive disorder^11^, social anxiety disorder^12^, and substance dependence^13^. Critically, ketamine has also been linked to the rapid alleviation of suicidal thoughts^14^, a particularly important unmet need given that suicide rates continue to rise in the United States and worldwide^15^. The RAAD literature has also expanded with the evaluation of other agents that target NMDAR antagonism, most notably the recent US Food and Drug Administration (FDA) approval of esketamine, the *S*-enantiomer of ketamine, as an adjunctive treatment for TRD^16^ and for adults with major depression with acute suicidal ideation or behavior. Off-label prescription of ketamine has also increased^17^. In part due to ketamine’s proven antidepressant effects, other substances with abuse potential that disrupt cognitive processes—including serotonergic psychedelics (SPs) such as psilocybin, Ayahuasca, lysergic acid diethylamide (LSD), and dimethyltryptamine (DMT), some of which appear to share downstream mechanisms with ketamine—are now also being re-evaluated as potential RAADs^18–22^. Taken together, this recent surge of neuropsychopharmacological research into ketamine and other RAADs has transformed our understanding of depression and treatment mechanisms.

Despite the considerable progress in this area, one key remaining question is the link between ketamine’s side effects and its antidepressant effects. In initial testing in the 1960s, individuals receiving ketamine described feeling “spaced out” or “dreaming” when administered subanesthetic doses^23^. Ed Domino, who was largely responsible for introducing ketamine into clinical practice as an anesthetic, described individuals who received ketamine patients as “disconnected from their environment somehow”^23^, prompting the coining of the phrase “dissociative anesthetic”^23^. Ketamine thus has a longstanding association with dissociation, broadly defined as altered consciousness and awareness of the self, environment, and reality.

In a landmark study, subanesthetic-dose ketamine (0.1 or 0.5 mg/kg) was studied in healthy volunteers as a proxy for schizophrenic psychosis^24^. It produced behaviors similar to the positive and negative symptoms and cognitive alterations seen in schizophrenia and evoked symptoms similar to dissociative states. Ketamine-induced psychotic symptoms included paranoia, tangentiality, loose associations, concreteness, ideas of reference, and unusual thought content^24^. At higher doses (0.5 mg/kg), derealization (i.e., feeling detached from surroundings), depersonalization (i.e., feeling detached from self), and altered perception of the body, environment, and time were all observed^24^. The findings suggested that ketamine produced a broad range of symptoms, behaviors, and cognitive deficits that resembled aspects of psychoses, particularly schizophrenia and dissociative states.

The focus of this manuscript, however, is subanesthetic-dose ketamine (0.5 mg/kg over 40 min) for the treatment of depressive symptoms. At this dose, systematic reviews have identified common side effects, including headache, dizziness, dissociation, elevated blood pressure, and blurred vision^25^. The dissociative side effects reported by individuals with depression included feeling strange, weird, bizarre, spacey, woozy, floating, or loopy^26,27^, reminiscent of the symptoms originally described by Domino^28^. Most side effects peaked within an hour of ketamine administration and resolved completely by 2 h post infusion. However, the specific relationship between ketamine’s dissociative effects and its immediate and longer-lasting antidepressant effects remains unknown. For instance, it is possible that ketamine’s dissociative symptoms are essential to the neurobiological mechanism of action required to produce antidepressant effects and, in fact, that ketamine’s antidepressant effects may depend on the experience of these side effects. Interestingly, psychedelic effects are an intrinsic aspect of treatment for some other agents that have abuse potential, such as psilocybin; such treatments often use psychological support before, during, and after administration to guide participants through the experience^18,21^.

This review will provide a brief overview of ketamine’s mechanism of action; consider the relationship between dissociative symptoms and antidepressant response to ketamine as well as the presence of dissociative symptoms in response to other NMDAR antagonists or modulators that have been evaluated as antidepressants; and offer future directions for research in this area, including preclinical models and in-depth biomarker work.

## Ketamine’s mechanism of action

An initial summary of ketamine’s proposed mechanism of action is necessary prior to exploring larger issues of its dissociative versus antidepressant effects. One hypothesis is that of NMDAR disinhibition. This theory postulates that antidepressant response to ketamine is initiated by the selective blockade of NMDARs on gamma aminobutyric acid (GABA)-ergic inhibitory interneurons. This, in turn, decreases interneuronal activity, which leads to disinhibition of pyramidal neurons and enhanced glutamatergic firing (“glutamate burst”). These processes then produce α-amino-3-hydroxy-5-methyl-4-isoxazole propionic acid receptor (AMPAR) activation, enhanced brain-derived neurotrophic factor (BDNF) release, tropomyosin receptor kinase B receptor activation, and subsequent promotion of protein synthesis via activation of the mechanistic target of rapamycin complex 1 of NMDARs^29^. Another theory gaining increased support is that ketamine blocks synaptic NMDARs involved in spontaneous synaptic transmission, which deactivates calcium/calmodulin-dependent kinase eukaryotic elongation factor 2 kinase, resulting in dephosphorylation of eukaryotic eEF2 and the subsequent de-suppression of BDNF protein synthesis in the hippocampus^30^. This signaling pathway then potentiates synaptic AMPAR responses, resulting in antidepressant efficacy.

Other theories for ketamine’s mechanism of action have been offered, but all propose changes in synaptic plasticity that lead to strengthening of excitatory synapses^31^ and share several characteristics and molecular mechanisms with well-characterized forms of homeostatic synaptic scaling^32^. Aspects of the disinhibition hypothesis were originally proposed by Moghaddam et al., who suggested that non-NMDAR hyperactivation—as opposed to “glutamatergic deficiency” from NMDAR blockade—might account for some of the cognitive deficits and schizophrenia-like symptoms associated with NMDAR antagonists^33^. This suggests that ketamine’s dissociative effects could be related to its mechanism of antidepressant action, as both appear to share important mechanistic processes. However, as discussed below, increasing evidence suggests that ketamine’s primary mechanism of antidepressant action is in fact NMDAR-independent^34^. While an in-depth evaluation of competing theories regarding ketamine’s mechanism of action is beyond the scope of this article, we refer the interested reader to other excellent reviews on this topic^30,32,35,36^.

## The correlation between dissociation and antidepressant response to ketamine

In 2014, Luckenbaugh et al. conducted a secondary data analysis of several potential predictors of antidepressant response to ketamine (*n* = 108)^37^. Neither change in manic or other psychotic symptoms nor change in systolic blood pressure, diastolic blood pressure, or pulse at 40 min post ketamine infusion were associated with later improvement in depressive symptoms. In contrast, dissociative symptoms, as measured by the Clinician-Administered Dissociative States Scale (CADSS)^38^, were associated with antidepressant improvement at both 230 min and 7 days post infusion. Although statistically significant, change in CADSS score from baseline explained only a small fraction of the variance in antidepressant response to ketamine^37^. In another study, “floating”—a depersonalization side effect (a type of dissociative side effect) did not mediate antidepressant response to ketamine assessed at 230 min post infusion and at day 1^27^. In addition, a systematic review of the eight published papers on this topic found that the relationship between dissociation and antidepressant effect was mixed; only three of the eight analyses observed a relationship between CADSS or other psychosis scores and antidepressant response^39^. Within the papers that reported a significant relationship, the explained variance of dissociative experiences for antidepressant response was 12–21%^39^. These mixed findings have been attributed to several factors.

First, it is possible that the relationship between dissociation and antidepressant effects may be a byproduct of functional unblinding. Given ketamine’s distinct side effect profile, unblinding over the course of clinical trials has been a longstanding concern^40,41^. However, it should be noted that even studies that limited unblinding—for instance by using blinded raters or active comparators such as midazolam—still found that ketamine had antidepressant effects^41^. Further arguments against functional unblinding include the variety of symptoms associated with ketamine administration^38^ and the fact that ketamine’s other side effects, such as blood pressure increases, were not associated with antidepressant effects. It is unlikely that participants, particularly those naive to drug experiences, would recognize dissociative experiences as indicative of ketamine administration as compared to changes in blood pressure or pulse. As noted above, the relationship between dissociative symptoms and antidepressant effects is inconsistent; if the association was purely due to unblinding, one would assume that the relationship would be more robust, given that most individuals treated with ketamine experience dissociation^26^.

Second, the trajectory of improvement is also important. Individuals with TRD usually receive an intravenous ketamine infusion over 40 min. Antidepressant effects and improvement in other symptom domains (e.g., anxiety, suicidal ideation) in response to a single ketamine infusion usually occur within a few hours and last ~1 to 2 weeks, peaking between 230 min and 1 day post infusion^2^. This pattern of onset and offset of antidepressant improvement has been consistently reported in numerous studies conducted at different sites around the world, reflecting what is likely a true biological effect rather than simple unblinding. Interestingly, the half-life of ketamine and its metabolite norketamine (NK) is only a few hours; thus, while significant improvement in depressive symptoms is observed within hours of drug administration, ketamine’s antidepressant and other therapeutic properties appear to last far beyond its half-life^42^.

Third, the CADSS was developed to measure dissociative effects before and after the recollection of traumatic memories in individuals with PTSD^38^. Thus, it is unclear whether this instrument is sensitive enough to measure the rapid changes in dissociative effects associated with investigational medications. A recent analysis of patients administered ketamine suggested that some dissociative experiences were not captured by the CADSS, including altered time and sensory perception, unusual bodily sensations, peacefulness, and disinhibition^43^. Other scales have been proposed to measure dissociative states in ketamine studies^44^, including the Altered States of Consciousness Rating Scale^45^, which assesses concepts such as “experience of unity”, “spiritual experience”, “blissful state”, or “disembodiment.” Such assessments might permit a more nuanced understanding of the ketamine experience. For now, however, it is possible that the mixed results surrounding ketamine’s antidepressant and dissociative symptoms might be attributable to imprecise measurement and to the difficulty of properly assessing individuals in an altered state.

Fourth, ketamine may have different effects in individuals with TRD compared to healthy volunteers. In one randomized, double-blind, placebo-controlled, crossover trial in which both participants with TRD (*n* = 35) and healthy volunteers (*n* = 25) were administered ketamine under the same conditions, the participants with TRD reported antidepressant effects, while the healthy volunteers demonstrated a transient increase in dysphoric symptoms^7^. The relationship between CADSS total score just after ketamine administration and worsening depressive symptoms in healthy volunteers did not reach statistical significance. If ketamine’s antidepressant effects were related to its dissociative side effects, it is unlikely that healthy volunteers would have such a divergent experience from individuals with TRD.

Finally, it is likely that if a relationship existed between ketamine’s antidepressant and dissociative effects, they would manifest in dose-finding studies. In one such study, participants with TRD were randomized to receive a range of single intravenous doses of ketamine (0.1, 0.2, 0.5, or 1.0 mg/kg) or an active control (midazolam)^6^. Although a relationship was observed between dose and dissociation—specifically, the higher ketamine doses (0.5 and 1.0 mg/kg) produced greater increases in CADSS scores than the lower doses—the higher dose did not result in greater acute antidepressant efficacy than the 0.5 mg/kg dose. Furthermore, no statistically significant correlations were found between CADSS scores 40 min post infusion and Hamilton Depression Rating Scale 6 (HAM-D-6) scores, the primary outcome measure^6^, although a 30-day follow-up analysis of individuals who had remitted or responded by day 3 found that the 1.0 mg/kg dose was associated with the lowest relapse rate^46^. This interesting finding warrants further investigation, as limitations of the study include a small sample size and the fact that concomitant medication use was allowed.

Taken together, the data reviewed above suggest little evidence for the notion that the acute antidepressant effects observed after a single ketamine infusion are directly due to its dissociative effects, although further studies examining long-term outcomes are needed. Differential findings between participants with TRD and healthy volunteers also signal the need to further explore the neurobiology of ketamine’s broad range of effects.

## Dissociation or other psychotomimetic effects associated with NMDAR antagonists and other related compounds

Compounds similar to ketamine represent another opportunity to evaluate the relationship between antidepressant and dissociative effects. If a drug is found to induce rapid antidepressant effects similar to those of ketamine without dissociative symptoms, this would suggest that the experiences are completely orthogonal—that is, that some other biological target is responsible for ketamine’s antidepressant effects that is separate from the target that induces dissociative symptoms. In contrast, if the only compounds that have demonstrated rapid antidepressant effects also cause psychotomimetic symptoms, this suggests that further examination of the role of dissociative experiences and depressive response are indicated, and that mixed results may be related to inadequacies associated with research design or methods. As noted above, the finding that ketamine exerts rapid antidepressant effects led to new avenues in drug discovery seeking to explore compounds with similar rapid effects. Because ketamine is an NMDAR antagonist, the bulk of initial drug development and discovery efforts focused on developing NMDAR antagonists that were therapeutically equivalent to ketamine but that did not have its side effects or abuse potential^47^. In comparison, research into other SPs embraced these compounds’ psychotomimetic experiences as integral to treatment (see below).

The first obvious comparison to ketamine is esketamine, the aforementioned *S*-enantiomer that recently received FDA approval as an adjunctive treatment for TRD. Esketamine is also associated with dissociative side effects; clinical trials found increased CADSS scores at 40 min post administration that resolved by 2 h, though concurrent increases in Brief Psychiatric Rating Scale (BPRS) positive symptom scores were not observed^48^. However, it should be noted here that intranasal esketamine is administered over 5–10 min, while intravenous ketamine is administered over 40 min. When repeated doses of esketamine were administered over induction, optimization, and maintenance phases, CADSS total scores were initially elevated after the first esketamine administration, but dissociative effects were generally attenuated or “flattened” over time during the maintenance of antidepressant response^49–52^. Therefore, while esketamine is associated with both dissociative and antidepressant effects, further evaluation of these effects and of differential response across acute, continuation, and maintenance treatment is needed, particularly with regard to long-term follow-up.

At least one agent was found to have a signal of antidepressant efficacy paired with profound dissociative effects. The compound, CP-101,606, is a selective NMDAR antagonist at the NR2B subunit and also has some sigma receptor effects. In a randomized, double-blind, placebo-controlled study of 30 participants with TRD, Preskorn et al. found that monotherapy with CP-101,606 had significant antidepressant effects that lasted up to 1 week^53^. However, during the trial, dissociative side effects were prominent and required both dose reductions and slower infusions, underscoring that NR2B receptor antagonists also have dissociative side effects. While antidepressant effects continued to be noted even after adjusting the maximum dose and rate of infusion, progress of this compound was eventually stopped due to side effect concerns.

Other NMDAR antagonists have provided more limited information regarding the link between dissociative and rapid antidepressant effects. One such class of agents is low-trapping NMDA channel blockers, such as AZD6765 (lanicemine). In theory, the greater trapping blockage in the NMDAR channel would lead to increased dissociative effects, while partial blockers would prevent the channel from closing, potentially leading to reduced dissociative effects^54^. Initial reports from smaller studies found that AZD6765 exerted immediate, short-lived, modest antidepressant effects with no difference in dissociative side effects from placebo^55,56^. However, a large Phase IIb study (*n* = 302) found that antidepressant effects did not separate from placebo^57^. Another low-to-moderate-affinity noncompetitive NMDAR antagonist is memantine, which is FDA-approved for the treatment of Alzheimer’s disease. While memantine did not have dissociative side effects, it also did not separate from placebo in the treatment of depressive symptoms^58,59^. Similarly, another orally administered NMDA NR2B antagonist, MK-0657 (CERC-301), neither produced dissociative side effects nor separated from placebo when evaluated in individuals with TRD^60,61^. In a double-blind, placebo-controlled study of 7-chlorokynurenic acid (7-Cl-KYNA), a potent and specific glycine site NMDAR antagonist, the investigators found that this agent had no antidepressant effects in TRD, nor were there differences in any of the peripheral or central biological indices or for adverse effects at any time between groups^62^. Lastly, several trials have investigated GLYX-13, an NMDAR glycine site functional partial agonist. In a proof-of-concept study, GLYX-13 was associated with rapid (within 2 h) reductions in depressive symptoms that lasted up to 2 weeks and no psychotomimetic side effects as assessed via the BPRS^63^. However, randomized, parallel-group Phase 3 trials found that GLYX-13 did not separate from placebo^64^.

As noted above, recent research with SPs such as psilocybin, LSD, DMT, and Ayahuasca suggest that these compounds may also lead to rapid antidepressant effects. Notably, the psychoactive state with SPs appears to be integral to the antidepressant effects of these agents; treatment thus often incorporates the psychedelic experience into assisted therapy^18^. In contrast, while there is a small literature on ketamine-assisted psychotherapy^65^, the preponderance of the ketamine research literature has conceived of psychotomimetic experiences related to ketamine as a side effect rather than a target to be explored in the context of treatment. While similarities in the mechanisms of action between SPs and ketamine are outside the scope of this article (see^22^ for a fuller examination), this burgeoning field may provide future insights into the relationship between psychotomimetic effects and the treatment of depression. For now, few of the cellular and molecular mechanisms implicated in ketamine’s mechanism of action have been systematically studied in SPs. Future evaluation of the antidepressant effects of SPs, particularly comparisons of antidepressant efficacy with and without psychological processing of the psychedelic experience, may help tease apart the cognitive and neurobiological processes involved in these treatments.

Taken together, the evidence reviewed above suggests that, except for a different formulation of ketamine (esketamine), no drug to date has been found to possess racemic ketamine’s rapid and robust antidepressant efficacy and its additional beneficial therapeutic profile for suicidal ideation, bipolar depression, anhedonia, and anxiety. As a result, no identified compounds have yet been shown to lead to ketamine-like antidepressant effects without dissociative symptoms, which precludes a unilateral dismissal of theories that dissociative and antidepressant experiences are related. While further evaluation of similar compounds, such as SPs, may provide valuable insight into this area, new approaches beyond current NMDAR inhibitors and related compounds may be needed to better understand ketamine’s mechanism of action, as detailed in the next section.

## Dissociative symptoms and RAADs: future directions

As noted above, the relationship between dissociative symptoms and rapid-acting antidepressant effects is inconclusive; specifically, no clear association has been observed between dissociative symptoms and antidepressant effects for ketamine, and no other NMDAR antagonists identified to date have demonstrated rapid antidepressant effects without dissociative symptoms. However, two distinct lines of inquiry may shed light on this putative relationship.

First, there is continuing pursuit of compounds seeking to replicate ketamine’s antidepressant effects without dissociative effects. In particular, several new avenues implicated in the mechanistic processes of ketamine’s antidepressant effects beyond NMDAR inhibition are now being explored that are likely to shed light on possible links between antidepressant response and dissociation. These include inhibition of extra-synaptic NMDARs, negative allosteric modulators of GABA receptors, group II metabotropic receptors (mGluR2/3) antagonists, blockade of spontaneous NMDAR activation, partial agonists and antagonists at the NMDAR complex, and inhibition of NMDA-dependent burst firing activity of lateral habenula neurons (reviewed in^66^). Another proposed mechanism suggests that ketamine’s antidepressant effects are NMDAR-independent and occur through ketamine’s metabolites; of these, (2*R*,6*R*)-hydroxynorketamine (HNK) is of particular interest^34,67^. Intriguingly, a recent study from our laboratory found that ketamine and NK plasma levels—but not (2*R*,6*R*)-HNK levels—were uniquely related to increased dissociative symptoms (as assessed via CADSS score) in participants with TRD^68^. Furthermore, the (2*R*,6*R*)-HNK metabolite may be particularly dependent on AMPARs and, thus, would not have dissociative side effects or abuse potential. Preclinical work as well as Phase 1 trials are ongoing to further evaluate whether the (2*R*,6*R*)-HNK metabolite can replicate ketamine’s antidepressant effects without its dissociative side effects. If one of these compounds can replicate ketamine’s antidepressant efficacy, questions regarding the role of dissociative effects would effectively be sidestepped, negating the need for further investigation.

Second, as briefly noted above, the currently inconclusive literature on dissociation may also be related to limitations associated with study design, particularly because most analyses have been post-hoc analyses of ketamine efficacy trials rather than studies designed to address the specific role of dissociation in antidepressant response. In this context, real-time assessments of biomarkers during ketamine administration may help address some of the limitations associated with self-report assessments of dissociative experiences. For instance, a growing literature exists regarding biomarkers of interest for antidepressant response to ketamine^69^, including gamma power, a marker of GABA system inhibition and excitatory glutamatergic neurotransmission, which has been shown to increase after ketamine administration^69,70^. In addition, magnetoencephalography (MEG) scans collected before and after ketamine administration have indicated that gamma power moderates ketamine’s antidepressant effects; specifically, for TRD participants with lower gamma power at baseline, increases in gamma power were associated with better antidepressant response^7^. Furthermore, resting-state functional magnetic resonance imaging (fMRI) studies found that default mode network connectivity within the insula was normalized in participants with TRD compared to healthy volunteers at the approximate peak of antidepressant response (day 2 post ketamine infusion), a change that was reversed 10 days post ketamine administration when participants were no longer displaying an antidepressant response^71^. Similarly, participants with TRD and healthy volunteers have also demonstrated paradoxical results on fMRI scans of emotional evaluation tasks after ketamine administration. For instance, post ketamine, participants with TRD had decreased blood oxygen level dependent activation in areas implicated in depression, while healthy volunteers had increased activation^72,73^. However, most of the biomarker work conducted to date uses markers collected hours to days after ketamine administration, well past the time points associated with ketamine’s dissociative effects and therefore not connected to specific dissociative experiences. In contrast, real-time data collection during ketamine administration, such as during MEG or fMRI^74^, could provide insights into the activation of specific neural circuits during dissociative experiences associated with ketamine administration. At the same time, cellular and molecular studies can provide additional insights into not only the mechanisms of RAADs but also dissociation and other psychotomimetic effects. Indeed, a recent study found that the dissociative effects of ketamine and PCP were associated with specific rhythms within layer 5 of the retrosplenial cortex in mouse models; similar rhythms were associated with dissociative symptoms in the posteromedial cortex of a human patient with focal epilepsy^75^. Such data could be further evaluated in light of specific theories related to ketamine’s mechanism of antidepressant action and its relationship to dissociative symptoms.

## Conclusions

Dissociation has been defined as “a discontinuity in the normal integration of consciousness, memory, identity, emotion, perception, body representation, motor control, and behavior”^76^. In the context of the evidence reviewed above, it remains unknown whether the dissociative experiences associated with ketamine administration represent a core feature of the antidepressant response or a side effect of a compound that will be minimized by future drug discovery efforts. To date, evaluation of other NMDAR-related compounds has not helped clarify this issue. Therefore, the relationship between dissociation and ketamine remains an open question. Future research should examine dissociation across preclinical models, neural systems, and cognitive processes in order to better understand their interplay and develop the most effective treatments for individuals with TRD while minimizing side effects
